# Parental perceptions of bilingualism and home language vocabulary: Young bilingual children from low-income immigrant Mexican American and Chinese American families

**DOI:** 10.3389/fpsyg.2023.1059298

**Published:** 2023-01-30

**Authors:** Emily Mak, Natasha Nichiporuk Vanni, Xintong Yang, Maria Lara, Qing Zhou, Yuuko Uchikoshi

**Affiliations:** ^1^School of Education, University of California, Davis, Davis, CA, United States; ^2^Department of Psychology, University of California, Berkeley, Berkeley, CA, United States

**Keywords:** dual language learners, home language and literacy environment, perceptions of bilingualism, oral proficiency, low-income, immigrant families

## Abstract

Dual language learners (DLLs), especially those from immigrant families in the United States, risk losing their home language as they gradually shift to speaking English as they grow up. Given the potential benefits of bilingualism on children’s cognitive, linguistic, and social–emotional development, it is crucial to maintain children’s home language to foster bilingual development. The current literature suggests that parental beliefs toward bilingualism and the language and literacy environment are linked to children’s language development. With the growing number of DLLs living in the United States, little is known about what parental beliefs about bilingualism of their children are integrated into these bilingual households and parents’ role in home language maintenance. The present study addresses the gap in the literature by investigating low-income immigrant families, specifically Chinese American and Mexican American families, and exploring the parental perceptions of children’s bilingual language learning. Further, the present study examines the relations among parental perceptions of bilingualism, home language and literacy practices, and home language oral proficiency. Data were collected from a total of 41 Mexican American and 91 Chinese American low-income immigrant families with DLLs ages 50–88 months who had been recruited from Head Start programs and state-funded preschools in Northern California when the children were 3–4 years old. Information about shared reading frequency, home language exposure and usage, and parental perceptions of bilingualism was collected through parental interviews, and DLLs’ home language oral proficiency was individually assessed. No significant difference in home language oral proficiency was observed between the two groups. Principal Components Analysis on the parental perceptions of bilingualism measure revealed two components, “Importance of Being Bilingual” and “English over Bilingualism.” Stepwise regression analysis results show that “Importance of Being Bilingual” was associated with children’s home language oral proficiency after controlling for culture, child age, the frequency of home language shared book reading, and child home language exposure and use. The results show that parents’ positive beliefs toward bilingualism are related to the children’s use of that language and their children’s language outcomes. Implications and suggestions for home language and literacy support for DLLs are discussed.

## Introduction

Dual language learners (DLLs) are defined as children who are learning two or more languages simultaneously at home (Espinosa, 2013). The rate of DLLs continues to grow, making up 32% of all children in the United States (Chung et al., 2019). An analysis of 26 states in the United States reported that DLLs make up 25.5% of their enrolled population in preschool programs, more than the general population (National Clearinghouse for English Language Acquisition, 2019). California reports that more than 40% of those enrolled in their state-funded preschool programs were DLLs (National Clearinghouse for English Language Acquisition, 2022). The predominant language spoken at home by DLLs is Spanish, accounting for three-quarters of DLLs’ early learning programs in California, followed by Mandarin and Cantonese, respectively (Brodziak et al., 2021). The percentage of DLLs who speak Chinese has grown by approximately 35% over the past eight years, and Chinese is the second most common home language in the United States (Batalova et al., 2021; Mitchell, 2020).

There are family factors that influence DLLs’ language and literacy development, such as parental involvement, family structure, and the quality of exposure to languages in the home (Portes and MacLeod, 1996). Findings also suggest that other demographic factors, such as parental education and socioeconomic status, influence the home language environment seeing as many DLLs live in poverty (Capps et al., 2005; Haft et al., 2021). Furthermore, parents of DLLs have varying acculturation beliefs, which may influence their choices in raising their bilingual children (Schwartz et al., 2010). DLLs, especially those from immigrant families, risk losing their home language as they enroll in schools and commonly use English (Nesteruk, 2010). Consequently, families may engage in different language and literacy practices to maintain their DLL children’s home language skills (Zhang and Slaughter-Defoe, 2009; García et al., 2012).

Few studies have investigated the relationship between parents’ beliefs toward bilingualism of their children and how that influences the home literacy environment and children’s bilingual attainment. Previous literature explains that bilingual development becomes conflictive when there are negative attitudes toward bilingualism and in some cases even the language itself; in such case, conflict instead of harmony in interpersonal interactions may result from subjective well-being (Veenhoven, 2008; De Houwer, 2013). The three-tier model of De Houwer (1999) describes how parents’ attitudes and beliefs, along with parents’ linguistic interactions and choices, result in the state of the child’s language development. To further examine these relationships, the present study examines parental perceptions of children’s bilingual language learning in low-income immigrant families, specifically Chinese American and Mexican American families.

## Theoretical framework

Previous research shows that parents play an essential role in a child’s language development (Taylor, 1983; García et al., 2012). A child’s first exposure to language occurs in the home, helping lay the foundation for the child’s literacy development. The family literacy theory states that the family is essential in developing the child’s emerging language and literacy skills (Taylor, 1983; García et al., 2022). Furthermore, parent involvement in their children’s learning and development has been found to positively impact academic achievement, frequently even more than the family’s socioeconomic status (Amatea, 2013). For DLLs, parental choice and frequency of language use influence their child’s bilingual development. As children enter schools in the United States, English becomes dominant in the child’s life, and home language exposure and development may only happen in the house.

Furthermore, the home literacy model states that children’s oral language and early literacy development are influenced by literacy activities at home (Sénéchal et al., 1998). Shared book reading allows parents to transfer knowledge and literacy skills to their children (Dexter and Stacks, 2014). A large body of research supports the positive effects of shared reading on children’s oral language outcomes, such as vocabulary and narrative skills (e.g., Sénéchal et al., 2008; Lever and Sénéchal, 2011; Malin et al., 2014; Lewis et al., 2016; Wasik et al., 2016; Gámez et al., 2017). More recently, the quantity of these opportunities, including the amount of language input, has been found to be associated with growth in the vocabulary of the two languages of bilinguals (Goodrich et al., 2021).

Moreover, Bronfenbrenner’s ecological systems theory suggests that the microsystem, which involves a child’s direct and immediate interactions with the environment and persons, including parents, siblings, teachers, and peers, serves as a proximal source for child learning and development (Bronfenbrenner, 2005; Goodrich et al., 2021). When children are young, they are mainly influenced by their home environment composed of their family members. Notably, parents have a significant effect on their children. In the home environment, parents expose children to the home language as well as the community language. Parents choose the home language and literacy environment with language activities and print opportunities that may assist in developing their vocabulary and understanding of language (Tunmer and Hoover, 1992).

## Parental perceptions of bilingualism and home language development

Parental beliefs toward bilingualism are linked to the language they use with their children at home and the school programs and language classes they let their children participate in (Wei, 2011). Many previous research articles show that the vast majority of Mexican American and Chinese American parents want their children to be bilingual and maintain their home language (e.g., Lao, 2004; Zhang, 2004, 2010; Scott, 2011; Portes and Rumbaut, 2014; Surrain, 2021; Hwang et al., 2022). Some reasons include heritage preservation, communication, and better career paths. Often, due to the lack of parental English abilities, immigrant parents cannot create a bilingual environment at home and are dependent on the schools to teach their children English (Oladejo, 2006; Chang, 2008).

Research has found that language practices at home can aid children’s language acquisition and development. Through a single mediator model, Ronderos et al. (2022) surveyed Spanish-English bilingual families and found a correlation between parental beliefs in Spanish leading to Spanish language outcomes and the same results for English. The ability to practice shared reading and language use at home could benefit the performance of that language outside of the home environment, making it easier to preserve the home language or grasp a new language. Children spend most of their time at school or with a parent. The parental perceptions of bilingualism as a positive trait can have a great influence on the success of fluent bilingual ability.

Recent research showed that most immigrant parents support bilingual education (Chang, 2008; Wei, 2011; Lau and Richards, 2021). Their reasons are focused on the hope that their children can develop a sense of national identity with their cultural roots, be able to communicate in the home language with older generations, and gain more job opportunities when they enter society (e.g., Tseng and Fuligni, 2000; Lao, 2004; Surrain, 2021; Hwang et al., 2022). Some parents only regard English as a tool; they think the home language is essential in forming meaningful relationships that maintain family ties (Oh and Fuligni, 2010). However, because children have lived in an English environment for a long time, they have spent more time systematically learning English. Some parents do not know how to create a home language environment for children to learn at home, which leads to their lack of vocabulary in the home language (Oladejo, 2006; Chang, 2008).

To become bilingual, DLLs’ home language needs to be maintained and developed, and family, particularly parents, play a significant role in home language maintenance in immigrant families (Guardado, 2002; Lutz, 2008; Brown, 2011; Park, 2013; Melo-Pfeifer, 2015). Research has shown that home language development is connected to children’s personal, cultural, and historical backgrounds and is vital for children’s development (Valdéz, 2001). Immigrant DLLs in the United States risk losing their home language as they gradually shift to speaking English more often when they begin school (Nesteruk, 2010; Portes and Rumbaut, 2014). Losing the home language may impair DLLs’ ethnic identity and even bonds with their families (Wong Fillmore, 2000; Lee, 2002; Oh and Fuligni, 2010; Ennser-Kananen, 2012; Mu, 2015). In addition, it may hinder DLL children’s relationships with their immigrant family members who speak only the home language (Qin, 2006).

Families also want to preserve their heritage and give their children the root of their culture (Zhang, 2010; Scott, 2011; Lee et al., 2015; Rosas, 2015; Surrain, 2021; Hwang et al., 2022). Parents state that heritage preservation is one of the reasons why bilingualism is advocated for, especially in families with home ties to their home countries (Farruggio, 2005). Because the older generation usually can only speak the home language, some parents report that they would feel embarrassed when their children cannot talk or understand the home language with the older generation in the family. Some parents only speak the home language, so they continue communicating with their children in the home language and hope their children will do the language brokering for them (Lee et al., 2015).

Parents of immigrant families put efforts into maintaining and instilling their DLL children’s home language skills as they are aware of the risk of home language attrition in their children (García et al., 2022). These parents generally have a positive attitude toward home language maintenance but may have different expectations and emphases in their children’s home language development (Liang, 2018). As the home language may be at risk in the absence of formal educational support, some parents enroll their children in home language education programs, while some employ home language policies at home and deliberately teach the language themselves (King et al., 2008; Curdt-Christiansen, 2009; Zhang and Slaughter-Defoe, 2009). One way of maintaining the home language is through shared book reading, where an adult reads a book with a child, exposes children to novel words, and transfers adults’ knowledge and literacy skills to the children (Wasik and Bond, 2001; Dexter and Stacks, 2014). Research has shown that shared book reading enhances children’s vocabulary knowledge (Wasik et al., 2016). Furthermore, a large body of research demonstrated the positive relations between parent–child shared book reading and language and literacy skills in young monolingual and bilingual children (e.g., Danis et al., 2000; DeTemple, 2001; Hindman et al., 2012; Leech and Rowe, 2014; Luo et al., 2021). These studies demonstrated that when parents engage their children in shared book reading by labeling, asking questions, and making comments, children are able to develop their language and literacy skills further. Often during shared book reading, parents discuss concepts uncommonly discussed in children’s daily lives and thus promote vocabulary and literacy skills in young children (Hindman et al., 2012). In addition, there is evidence that the home language use during parent–child shared book reading promotes home language development among bilingual preschoolers (e.g., Sun, 2019; Sun et al., 2022a).

In addition, bilingual practices and opportunities for exposure may depend on the family’s socioeconomic status. Immigrant parents may be unable to support the new language due to their own language barriers (Leyendecker et al., 2018). It was found that low-income families use their home language more than the English language with their children (Williams et al., 2019; Haft et al., 2021). Therefore, home language input may vary across families for these DLL children. Moreover, home language input from parents was found to be positively related to young DLLs’ home language outcomes (Dixon et al., 2012; Mori and Calder, 2017; Sun et al., 2020). Language input and output should be treated equally as a learning process for DLL children. According to the input hypothesis, language input is traditionally seen as a key component for children when learning a new language (Krashen, 1985). However, recent research has begun recognizing children’s language output as another unique contributor to bilingual language development (e.g., Bohman et al., 2010; Bedore et al., 2016; Ribot et al., 2018). The importance of language output in bilingual language learning is supported by the output hypothesis (Swain, 2005). According to the output hypothesis, other than language exposure, being able to produce the target language actively and getting confirmation or negative feedback from more proficient speakers are vital to learning a second language. A recent study showed that that both home language input and output of the child significantly predicted the home language proficiency in bilingual kindergarteners (Sun et al., 2022b). Thus, the relationships between DLLs’ language input and output and oral language proficiency are examined in this present study. Most previous studies on DLLs’ home language and literacy have focused on English language outcomes. More research is needed on DLL children’s home language development and their language and literacy environment, including the quantity of language input and output.

## Present study

Given the benefits of maintaining the home language for DLLs’ development, the present study aimed to investigate the relationships among parental perceptions of bilingualism for their child, home language and literacy environment, and home language vocabulary outcomes among low-income immigrant DLL children of Mexican American and Chinese American families. First, the associations between perceptions of bilingualism and the home language and literacy environment were examined. It was hypothesized that parental perceptions of bilingualism would be related to children’s home language and literacy environment. Next, the associations between perceptions of bilingualism, home language and literacy environment, and home language vocabulary were examined. It was expected that parents’ perceptions of bilingualism and the home language and literacy environment would be positively associated with DLLs’ home language vocabulary.

## Materials and methods

### Participants

A total of 132 DLLs from low-income immigrant families were recruited from Head Start centers and state-funded preschools in Northern California from Fall 2018 to Fall 2019 (Time 1). These children were ages 3–4 when they were recruited through parent meetings and drop-off times during regular school hours at Time 1. Follow-up data collection was conducted 1.5 years later, from Fall 2020 to Fall 2021 (Time 2). Data from Time 2 will be used and discussed in this study.

At Time 2, Mexican American DLLs’ ages ranged from 50 to 88 (*M* = 67.19; *SD* = 9.18), and Chinese American DLLs’ ages ranged from 52 to 88 months (*M* = 71.27; *SD* = 5.89). 46.34% of the Mexican American DLLs and 56.04% of Chinese American DLLs were boys. The average Mexican American maternal educational years was 11.76 years (range = 8–18; *SD* = 3.08), and the average Chinese American maternal educational years was 12.76 years (range = 8–18; *SD* = 2.40). The average Mexican American family *per capita* income in the previous year, calculated by total family household income divided by household size, was US$9,320.41 (range = US$1,500–$24,375; *SD* = US$5,390.98), and the average Chinese American family *per capita* income in the previous year was US$9,634.20 (range = US$1,000–$29,166.67; *SD* = US$6,572.93).

### Measures

#### Parental perceptions of bilingualism

Parental perceptions of bilingualism for one’s child were measured using the Perceptions of Bilingualism for Child Plus scale (PoB+; Luk and Surrain, 2019). This eight-item scale was designed to measure parents’ perceptions of the value of bilingualism for their DLL children. The eight questions asked parents about the benefits and potential costs of bilingualism for their children, with two items being reverse-coded. Each question was translated into Spanish and Chinese. The questions were asked on a six-point Likert scale from 1 (strongly disagree) to 6 (strongly agree). The Cronbach’s alpha of the eight questions was 0.77. See Table 1 for a list of questions. The inverse of the reverse coded items (Questions 5 and 7) of the PoB+ was used.

**Table 1:** List of perceptions of bilingualism for child plus scale questions, means, and standard deviations (*N* = 132).

		*M*	*SD*
1	It is important for my child to speak more than one language.	5.50	1.07
2	Speaking more than one language will help my child succeed in school in the long term.	5.53	0.90
3	It is important for my child to learn to read and write more than one language.	5.61	0.75
4	Speaking more than one language will help my child compete in the job market.	5.66	0.71
5	My child will be confused if he or she learns two languages at the same time.	4.27	1.50
6	Speaking more than one language will help my child become a stronger thinker.	4.94	1.26
7	To be successful, the only language my child needs to speak well is English.	4.76	1.21
8	Speaking more than one language will help my child understand people from different cultural backgrounds.	5.41	1.02

M, mean; SD, standard deviation; items 5 and 7 were reverse coded.

#### Parental language input and child language output

The parental language input and child language output questionnaire was adapted from the Bilingual Input–Output Survey (BIOS; Peña et al., 2014). Parents reported on the hour-by-hour language input and the child’s language output of English and the home language in the home for any typical weekdays and weekends. The relative percentages of hours of home language parent input and child output compared to English were used for data analysis.

#### Shared reading frequency

Parents reported on the shared reading frequency in the home language with their child in the home on a six-point Likert scale (0 = *never*, 1 = *once a month*, 2 = *2–4 times a month*, 3 = *once a week*, 4 = *2–3 times a week*, and 5 = *every day*) adapted from Hammer et al. (2003).

#### Home language vocabulary

Home language vocabulary was measured by the Vocabulario Sobre Dibujos (Picture Vocabulary) subtest from the *Woodcock-Johnson, 4th Edition, Tests of Oral Language* (Schrank et al., 2014). In this task, children were asked to identify objects presented to them in pictures by providing single-word answers in their home language (i.e., Spanish or Chinese). There were 54 test items in total. All children started from the first item and stopped when they responded incorrectly to the last six items consecutively. The median test reliability for Spanish at age 6 is 0.88 (Wendling et al., 2019).

The Chinese version of the Picture Vocabulary subtest was translated from the Spanish subtest and verified by language experts, which has been done in previous studies (e.g., Uchikoshi, 2013; Chung et al., 2019; Chernoff et al., 2021; Uchikoshi et al., 2022). The alpha reliabilities in Chinese for our sample was 0.90. Raw scores were used for both Mexican American and Chinese American participants as there were no standardized scores for the Chinese American population in the United States.

### Data analysis

First, Shapiro–Wilk tests were run to check for normality for all variables. Then, Mann–Whitney tests were used to compare non-normally distributed dependent variables between the Mexican American and Chinese American groups. Kendall’s correlations were conducted to examine the relations between all variables. The PoB+ data were also examined and evaluated with Principal Component Analysis. Finally, stepwise regression analysis was used to examine the unique associations between parental perceptions of bilingualism and children’s home language proficiencies. The descriptive statistics were computed using RStudio Version 1.4.1717 (RStudio Team, 2021), and the Principal Component Analysis and stepwise regression analysis were conducted using IBM SPSS Statistics for Macintosh, Version 28.0 (IBM Corp, 2020).

## Results

The descriptive statistics of the study variables are presented in Table 2. The average frequency of parent–child shared book reading in the home language was 2.71, which can be interpreted to be roughly less than once a week. The standard deviation was 1.86, meaning there were variations in how often the parents read to their children in our sample. Some parents never read, while some read every day. The average percentages of parent home language input and child home language output were 49 and 46%, respectively. This indicates an equal amount of home language and English was spoken in the home of these immigrant families. The mean raw score of Picture Vocabulary in the home language was 14.62 with some variations (range = 0–30; *SD* = 6.94). No significant difference in Picture Vocabulary scores was observed between the Mexican American and Chinese American groups. Table 3 presents the correlations between all study variables. Parent home language output and shared reading frequency in the home language were positively correlated with Picture Vocabulary in the home language.

**Table 2:** Descriptive statistics of study variables.

	*N*	*M*	*SD*	Min	Max	Skewness	Kurtosis
Child Age (Months)	128	70.02	7.27	50	88	−0.34	0.67
HL Shared Reading	132	2.71	1.86	0	5	−0.35	−1.43
Parent HL Input	128	0.49	0.19	0	1	0.03	0.11
Child HL Output	128	0.46	0.22	0	1	0.02	0.18
HL Picture Vocabulary	122	14.62	6.94	0	30	−0.22	−0.50

M, mean; SD, standard deviation; and HL, home language. Raw scores were used for picture vocabulary.

**Table 3:** Correlation among the study variables.

Variables	1	2	3	4	5	6	7	8
1. Culture	–							
2. Child Age	−0.26^**^	–						
3. Parent HL Input	−0.07	−0.04	–					
4. Child HL Output	−0.08	−0.08	0.76^***^	–				
5. Importance of Being Bilingual	−0.25^**^	−0.01	−0.06	−0.05	–			
6. English over Bilingualism	−0.44^***^	−0.33^***^	0.09	0.11	0.00	–		
7. HL Shared Reading	−0.25^**^	0.001	0.11	0.10	0.26^**^	0.11	–	
8. HL Picture Vocabulary	−0.07	0.18^*^	0.11	0.19^*^	0.33^***^	0.07	0.36^***^	–

HL, home language; ^*^p < 0.05, ^**^p < 0.01, and ^***^p < 0.001.

### Parental perceptions of bilingualism

Mean values of the PoB+ are stated in Table 1. Questions 1, 2, 3, 4, and 8 all had mean scores of around 5.5 on a scale of 1 (strongly disagree) to 6 (strongly agree). Question 6 was slightly under 5, indicating that the majority of parents agreed. In general, parents believed that it was important for their children to become bilingual and biliterate to succeed in school and compete in the job market, as well as become more culturally competent and strong thinkers.

The factorability of the PoB+ data was examined. All eight items correlated at least 0.3 with at least one other item, suggesting reasonable factorability. Principal Components Analysis with varimax rotation on the eight items indicated two components with an Eigenvalue >1. The Kaiser-Meyer-Olkin measure of sampling adequacy was 0.84, which is higher than the recommended value of 0.6. Further, Bartlett’s test of sphericity was significant [χ^2^(28) = 443.65, *p* < 0.001]. Six items loaded onto Component 1: (item 1 importance of being bilingual, item 2 success in school, item 3 importance of being biliterate, item 4 success in the job market, item 6 stronger thinker, and item 8 understanding other cultures). Two items loaded on Component 2 (item 5 confusion between two languages and item 7 English over other languages). The first principal component addressed parents’ perception of the “Importance of Being Bilingual,” and the second principal component addressed parents’ perception of “English over Bilingualism.” Internal consistency for both of the scales was examined using Cronbach’s alpha. The alphas were 0.86 for “Importance of Being Bilingual” (six items) and 0.56 for “English over Bilingualism” (two items).

### Stepwise regression analysis

To account for possible multicollinearity effects among variables, stepwise regression analyses were conducted. The model was specified to determine associations among parental perceptions of bilingualism for their child, home language and literacy environment, and home language vocabulary outcomes. Specifically, the two components of parental perceptions of bilingualism (“Importance of Being Bilingual” and “English over Bilingualism”), culture, child age, home language exposure and usage, and home language reading frequency were entered into the model to find the best fitting model. The estimates of the unstandardized regression coefficients, standardized regression coefficients, and *R^2^* values of the final model are reported in Table 4.

**Table 4:** Stepwise regression analysis predicting home language oral proficiency.

	*B*	β	*SE*
Importance of Being Bilingual	1.92^***^	0.28	0.58
HL Shared Reading	0.99^**^	0.27	0.31
Child HL Output	6.36^*^	0.20	2.59
Child Age (Months)	0.19^*^	0.20	0.08
Constant	−4.22		5.67
*R^2^*		0.26	
Adjusted *R^2^*		0.24	
*F*		10.16^***^	

HL, home language; B, unstandardized regression coefficients; β, standardized regression coefficients, SE = standard error; ^*^p < 0.05, ^**^p < 0.01, and ^***^p < 0.001.

The final model explained 23.5% of the variance in home language oral proficiency as measured with Picture Vocabulary. “Importance of Being Bilingual” was associated with home language oral proficiency. “English over Bilingualism” was not associated with home language oral proficiency. As predicted, child age was also associated with home language oral comprehension. An increase in age was associated with higher home language oral proficiency. Home language output was also associated with home language oral proficiency. The more the child used the home language at home, the higher the oral proficiency skills were. Furthermore, home language reading frequency was associated with home language oral proficiency. The more frequently the parent and the child read a book together in the home language, the higher the child’s home language oral proficiency skills were.

## Discussion

The purpose of this study was to examine the associations among parental perceptions of bilingualism for their child, home language and literacy environment, and home language oral proficiency in low-income immigrant DLL children of Mexican American and Chinese American families. The results indicated that Mexican American and Chinese American immigrant parents’ beliefs, along with shared reading practices and children’s language use, are related to DLL children’s oral proficiency in the home language.

### Immigrant parents’ perceptions of bilingualism

The Principal Components Analysis of the eight-item PoB+ data revealed two factors that represented parental perceptions toward their children’s bilingualism which was the “Importance of Being Bilingual” and “English over Bilingualism.” However, only the former was associated with children’s home language oral proficiency. Learning English is favored due to it being a majority language in the United States (Ronderos et al., 2022), but with the next most spoken languages in the nation being Spanish and Chinese during the years 2005–2019 (US Census Bureau, 2019), being biliterate serves as an academic, social, professional, and cultural advantage.

Aligned with previous research, our findings also revealed that parents believed that bilingualism equips children to become more competitive in the job market. Both Mexican American and Chinese American families view bilingual attainment as a precursor to better career opportunities (Lao, 2004; Surrain, 2021). A possible explanation lies in the existing thought that maintaining a minority language and developing proficiency in a majority language will lead to increased economic opportunities (McCabe et al., 2013), with many Latino families believing that the Spanish and English languages are essential for success in the United States (Taylor et al., 2012). Many employers are now looking to hire bilingual or multilingual individuals, for they know that the demand for service in various languages is continuously increasing. This trend can be observed by examining demographic trends in the United States. It is projected that in the year 2030, net international migration will introduce 1.1 million people to the population, more than the nation’s natural increase, and the trend will continue for the following years (Vespa et al., 2018). Given this, customers, employers, and companies will benefit from bilingual employees, hence increasing job opportunities for bilingual individuals.

Another finding showed that immigrant Mexican American and Chinese American families encouraged bilingualism so that their children could develop cultural competence. Consistent with previous research, parents associate children losing their home language with losing connections to one’s cultural identity and community (Imbens-Bailey, 1996; Pease-Alvarez, 2003). When children and family are equally able to communicate in their home language, family closeness and values are maintained, whereas if communication between the parties is difficult, then conflict, perceived distance, and disagreements are more common in Asian Pacific and Latin American families (Shon and Ja, 1983; Tseng and Fuligni, 2000). For children from immigrant families, home language proficiency is essential for supporting ethnic identity and parent–child relationships (Oh and Fuligni, 2010). Overall, bilingualism for children of immigrant families is helpful for psychosocial and emotional well-being.

Furthermore, our findings revealed that parents believed bilingualism would help their children become strong thinkers. Access to two languages and cultures naturally exposes children to a wide range of experiences, perspectives, and beliefs (Poarch and Krott, 2019). These opportunities shape bilingual children’s cognitive and social development and allow bilingual children to become more open-minded and develop cultural empathy from a young age (Poarch and Krott, 2019). These aspects of social cognition are important in developing friendships, communicating with peers and teachers, and understanding text. As a result, bilingual children may become strong thinkers. Moreover, since bilingual children are exposed to various perspectives and beliefs from a young age, recent research suggests bilingual preschoolers have less implicit racial bias when compared to their monolingual peers (Singh et al., 2020).

### Parental perceptions of bilingualism and home language development

The regression results of this study revealed significant associations between parental perceptions of bilingualism on the “Importance of Being Bilingual,” home language and literacy environment, and home language oral proficiency. Aligned with the existing literature, the results showed that parents’ positive beliefs with regard to maintaining a target language increase the use of that language and improve their children’s language outcomes (De Houwer, 1999; Ronderos et al., 2022). DLLs with parents who believe that maintaining the home language and societal language, English, is essential to have higher oral proficiency in their home language. Past studies have demonstrated that most Mexican American and Chinese American parents are determined to maintain the home language when raising their DLL children (e.g., Lao, 2004; Zhang, 2010; Scott, 2011; Lee et al., 2015; Hwang et al., 2022). Immigrant parents believe that the home language represents family, childhood, heritage, and culture, while the societal language represents education, career, and opportunities (Edgerton and Karno, 1971; Lao, 2004; Zhang, 2010; Surrain, 2021). Some of these parents encourage their children to maintain the home language at home and acquire the societal language in school, as bilingualism is seen to be essential to be successful (Taylor et al., 2012).

In addition, the results demonstrated a positive association between child home language use and home language oral proficiency, which aligns with the output hypothesis (Swain, 2005). Children who use more home language with their parents achieve greater home language oral proficiency. This finding supports the previous findings with regard to language use as a learning process in language development (Hammer et al., 2009; Bohman et al., 2010; Bedore et al., 2016; Ribot et al., 2018). A possible explanation why language production is key to language growth is that the process involved in talking differs from hearing. Producing words challenges children’s linguistic systems to respond and allows children to practice the mechanism of retrieval (Bohman et al., 2010; Rowe et al., 2017; Ribot et al., 2018). This allows DLLs to practice their home language and thus improve their oral proficiency.

Contrary to the existing literature in which language exposure plays a role in shaping children’s language development (Hoff, 2018), parent home language input was found to be not related to DLLs’ home language oral proficiency. A plausible explanation of this finding is that the quantity of language exposure alone was not enough for DLL children to develop proficiency in the home language. Although previous studies suggest that the quantity of home language exposure at home was related to DLLs’ home language vocabulary outcomes (e.g., Branum-Martin et al., 2014; Cheung et al., 2019), the quality of language exposure also plays a role in children’s language outcomes. Rowe and Snow (2020) state that the quality of language exposure in early childhood development matters in children’s language earning trajectory. The quality of language exposure is characterized by having the opportunities to have back-and-forth communication, exposure to novel and sophisticated vocabulary, and challenges through inferential discussion. Future research should consider investigating the quality of language exposure in addition to the quantity of exposure.

Another significant finding from this study is that shared book reading in the home language has a positive effect on children’s home language oral proficiency. This finding supports the previous studies that shared book reading is positively associated with English language outcomes (e.g., Hindman et al., 2012; Leech and Rowe, 2014) and home language outcomes (Cheung et al., 2019; Paradis et al., 2021; Ronderos et al., 2022). Reading to a child allows parents to teach and transfer knowledge to their children (Dexter and Stacks, 2014). Therefore, it is an excellent opportunity for DLLs to be exposed to the home language, learn vocabulary, and have discussions that otherwise would not occur in their daily lives, especially when they receive education in primarily the societal language, English.

### Limitations and future directions

The current study provides evidence that parents’ beliefs and home language and literacy environment contribute to DLLs’ home language oral proficiency. However, some limitations should be taken into account. First, in addition to examining the quantity of language practices, it would be desirable to investigate the quality of parent home language input and child language home output to identify the mechanisms responsible for the effects on DLLs’ home language outcomes. Furthermore, future research should consider examining the mediating effects of home language and literacy practices to understand further the relationships between parent beliefs, home language and literacy environment, and child language outcomes. As demonstrated in a previous study, the association between parent beliefs and children’s language outcomes may be mediated by children’s choice of language use (Ribot et al., 2018). Moreover, this study only explored DLLs’ expressive vocabulary and not receptive vocabulary due to time constraints. Future research should examine both expressive vocabulary and receptive vocabulary. It is also important to note that the home language oral proficiency assessment tool used in this study was only normed with Spanish-English bilingual children. Having an appropriate assessment tool for the Chinese-English bilingual children would be essential to assess these children’s Chinese oral language proficiency accurately. Since the sample of the current study was from low-income households, future researchers could investigate the relationship between parent beliefs toward children’s bilingualism and DLLs’ home language outcomes of families with high socioeconomic status and explore potential differences between low and high socioeconomic status families.

## Conclusion

As DLL children of immigrant families in the United States acquire English, they may face challenges in maintaining their home languages and gradually lose their home language skills as they begin school (Paradis et al., 2021). Our study confirmed the vital role of parents in the minority home language development of these young DLLs. The findings suggested that immigrant parents who believe in the importance of bilingualism and employ particular home language and literacy practices, including reading to their children and allowing their children to use the home language, often lead to more positive outcomes for DLLs’ home language proficiency. Parents who wish their children to maintain bilingualism successfully should engage in literacy practices in the home language to promote home language development in their children. It is also essential to encourage their children to practice producing the home language. Having a balanced usage of the home language and English would be ideal and sufficient to avoid home language retention as these children age.

## Data availability statement

The raw data supporting the conclusions of this article will be made available by the authors, without undue reservation.

## Ethics statement

The studies involving human participants were reviewed and approved by University of California, Berkeley IRB and University of California, Davis Reliance IRB. Written informed consent to participate in this study was provided by the participants’ legal guardian/next of kin.

## Author contributions

EM, NN, XY, ML, QZ, and YU contributed to the conception and design of the study, writing and revision of the manuscript, and approval of the submitted version.

## Funding

This study was supported by the Eunice Kennedy Shriver National Institute of Child Health and Human Development of the National Institutes of Health under award number R01HD091154.

## Conflict of interest

The authors declare that the research was conducted in the absence of any commercial or financial relationships that could be construed as a potential conflict of interest.

## Publisher’s note

All claims expressed in this article are solely those of the authors and do not necessarily represent those of their affiliated organizations, or those of the publisher, the editors and the reviewers. Any product that may be evaluated in this article, or claim that may be made by its manufacturer, is not guaranteed or endorsed by the publisher.
